# Development and External Validation of an Updated Nomogram Including Both MRI and Local PSMA PET/CT Information for the Prediction of Side-specific Extraprostatic Extension in Patients Treated with Radical Prostatectomy

**DOI:** 10.1016/j.euros.2026.10.003

**Published:** 2026-10-29

**Authors:** Vera Sweere, Alexandra Bruins Slot, Joris G. Heetman, Rick Hermsen, Lieke Wever, Jules Lavalaye, Maarten Vinken, Clinton D. Bahler, Mark Tann, Claudia Kesch, Tugce Telli, Wolfgang P. Fendler, Peter Ka-Fung Chiu, Sam Kwan Kit Wu, Fabio Zattoni, Laura Evangelista, Francesco Ceci, Marcin Miszczyk, Paweł Rajwa, Akihiro Matsukawa, Tamás Fazekas, Francesco Barletta, Alberto Briganti, Giorgio Gandaglia, Jean-Paul A. Van Basten, Harm H.E. Van Melick, Roderick C.N. Van den Bergh, Giancarlo Marra, Matthijs J.V. Scheltema, Timo F.W Soeterik

**Affiliations:** a Department of Urology, St. Antonius Hospital, Nieuwegein/Utrecht, The Netherlands; b Department of Urology, Amsterdam University Medical Center, Amsterdam, The Netherlands; c Department of Nuclear Medicine, Canisius Wilhelmina Hospital, Nijmegen, The Netherlands; d Department of Nuclear Medicine, St. Antonius Hospital, Nieuwegein/Utrecht, The Netherlands; e Department of Urology, Indiana University Medical Center, Indianapolis, IN, USA; f Department of Radiology and Imaging Sciences, Indiana University Medical Center, Indianapolis, IN, USA; g Department of Urology, University Hospital Essen, Essen German Cancer Consortium (DKTK) University Hospital Essen, Essen, Germany; h Department of Nuclear Medicine, University Hospital Essen, Essen German Cancer Consortium (DKTK), Germany; i S. H. Ho Urology Centre, Department of Surgery, The Chinese University of Hong Kong, Hong Kong, China; j Department of Nuclear Medicine and PET, Hong Kong Sanatorium and Hospital, Hong Kong, China; k Department of Surgery, Oncology, and Gastroenterology, Urological Unit, University of Padova, Padova, Italy; l Department of Biomedical Sciences, Humanitas University, Pieve Emanuele, Milan, Italy; m Division of Nuclear Medicine, IRCCS Humanitas Research Hospital, Milan, Italy; n Division of Nuclear Medicine and Theranostics, IEO European Institute of Oncology, IRCCS, Milan, Italy; o Department of Urology, Comprehensive Cancer Center, Medical University of Vienna, Vienna, Austria; p Collegium Medicum–Faculty of Medicine, WSB University, Dąbrowa Górnicza, Poland; q Department of Urology, Medical University of Silesia, Zabrze, Poland; r Division of Surgery & Interventional Science, University College London, London, UK; s Department of Urology, The Jikei University School of Medicine, Tokyo, Japan; t Department of Urology, Semmelweis University, Budapest, Hungary; u Division of Oncology/Unit of Urology, Soldera Prostate Cancer Lab, Urological Research Institute (URI), IRCCS San Raffaele Scientific Institute, Vita-Salute San Raffaele University, Milan, Italy; v Department of Urology, Canisius Wilhelmina Hospital, Nijmegen, The Netherlands; w Department of Urology, Erasmus Medical Center, Rotterdam, The Netherlands; x Division of Urology, Department of Surgical Sciences, Molinette Hospital, Città della Salute e della Scienza and University of Turin, Italy; y Department of Radiation Oncology, University Medical Center Utrecht, Utrecht, The Netherlands

**Keywords:** Extra prostatic extension, MRI, Prostate cancer, PSMA PET/CT

## Abstract

**Background and objective:**

The predictive value of prostate-specific membrane antigen (PSMA) positron emission tomography/computed tomography (PET/CT) for extraprostatic extension (EPE) in prostate cancer remains unclear.

**Objective:**

We assessed whether adding PSMA PET/CT to magnetic resonance imaging (MRI) and clinical factors improves side-specific EPE prediction.

**Methods:**

Patients undergoing PSMA PET/CT and MRI before radical prostatectomy were analysed retrospectively. The endpoint was side-specific EPE (≥pT3a). Predictors were selected by backward elimination, and the final nomogram was fitted using generalised estimating equations to account for clustering of lobes within patients. Discrimination, calibration, and net benefit were assessed, with external validation in an independent single-centre cohort.

**Results and limitations:**

Development and validation cohorts included 801 and 287 lobes. The final model comprised prostate-specific antigen density (odds ratio [OR] 1.54 [95% confidence interval {CI} 0.94–2.50]; biopsy International Society of Urological Pathologists grade group (2 vs benign/1: OR 2.01 [95% CI 1.27–3.19]; 3 vs benign/1: OR 3.82 [95% CI 2.30–6.35]; 4 vs benign/1: OR 2.50 [95% CI 1.54–4.08]; 5 vs benign/1: OR 4.05 [95% CI 2.18–7.53]), MRI T-stage (organ-confined with a lesion [OC] vs organ-confined with no lesion [NL]: OR 1.31 [95% CI 0.86–1.99]; EPE vs NL: OR 4.11 [95% CI 2.56–6.60]), PSMA T-stage (OC vs NL: OR 1.67 [95% CI 1.07–2.61]; EPE vs NL: OR 2.59 [95% CI 1.49–4.49]), PSMA_total_ (OR 1.72 [95% CI 1.33–2.23]). In development, the PSMA-MRI nomogram showed superior discrimination (area under the receiver operating characteristic curve [AUC] 0.80 vs 0.71, *p* < 0.001). On validation, the updated model demonstrated discrimination comparable to the reference model (AUC 0.85 vs 0.86, *p* = 0.9), with minor risk overestimation and similar net clinical benefit. Limitations include a relatively small validation cohort.

**Conclusions:**

The PSMA-MRI nomogram appeared to outperform the MRI-only model in development, but this was not confirmed in validation, indicating limited, context-dependent incremental value.

## Take Home Message

A combined prostate-specific membrane antigen positron emission tomography/computed tomography (PSMA PET/CT)–magnetic resonance imaging (MRI) model improved extraprostatic extension prediction in development but showed similar performance to MRI in external validation. The added value of PSMA PET/CT appears context-dependent and warrants further investigation.

ADVANCING PRACTICE **What does this study add?**

A prostate-specific membrane-antigen positron emission tomography/computed tomography (PSMA PET/CT)–magnetic resonance imaging (MRI) nomogram may enhance prediction of side-specific extraprostatic extension beyond MRI alone in selected settings. However, comparable performance on external validation highlights that the incremental clinical value of PSMA PET/CT may depend on patient population and imaging context

**Clinical Relevance**

Accurate prediction of extraprostatic extension (EPE) is essential for treatment planning, particularly when deciding between nerve-sparing surgery and wider excision or alternative treatment strategies. This study suggests that integrating PSMA PET/CT with MRI and clinical factors may improve side-specific prediction of EPE, although its incremental value over MRI alone was not consistently demonstrated on external validation. These findings highlight the importance of using all available imaging modalities in a complementary and evidence-based manner to optimise prediction of EPE and support individualised treatment decision-making. Editor-in-Chief: Jochen Walz.

**Patient Summary**

In this study, we evaluated whether adding prostate-specific membrane antigen positron emission tomography/computed tomography (PSMA PET/CT) to magnetic resonance imaging (MRI) improves the prediction of tumour spread outside the prostate. We found that combining both imaging techniques improved predictions in the initial patient group but showed similar performance to MRI alone in an independent group. These findings suggest that the added value of PSMA PET/CT may depend on the clinical setting, and further research is needed to better define its role.

## 1 Introduction

Accurate assessment of the presence of extraprostatic extension (EPE) of prostate cancer (PCa) is crucial in determining the optimal treatment approach [1]. Although radical prostatectomy (RP) offers good oncological control, surgery is associated with an increased risk of urinary incontinence and erectile dysfunction [2]. Therefore, nerve-sparing techniques have been introduced, substantially lowering the risk of postoperative incontinence and erectile dysfunction, although the quality of evidence is low [3]. However, performing nerve-sparing may be associated with an increased risk of ipsilateral positive surgical margins [4], [5]. Because both EPE and surgical margin status after RP are associated with biochemical recurrence risk, patients should be carefully selected for nerve-sparing approaches [6].

Magnetic resonance imaging (MRI) is an effective modality to assist local staging of PCa. However, its sensitivity for the detection of EPE remains suboptimal, with detection rates reported between 51–57% [7], [8]. To enhance EPE risk prediction, MRI findings are frequently combined with other clinical parameters [9], [10]. In recent years, several EPE nomograms incorporating MRI data have been developed [11], [12], [13]. Among these, the nomogram described by Soeterik et al, including side-specific MRI tumour staging, biopsy International Society of Urological Pathology (ISUP) grade group (GG), and prostate-specific antigen (PSA) density (PSAD; model 2), has been validated in multiple studies and has demonstrated consistent accurate discrimination and calibration [11], [14], [15], [16].

Recently, the Next Generation Trial showed that prostate-specific membrane antigen (PSMA) positron emission tomography/computed tomography (PET/CT) had superior local tumour (T)-staging compared to MRI [17]. In addition, Li et al reported that combining PSMA PET/CT with MRI showed increased sensitivity for detecting EPE [18]. Another study demonstrated that PSMA PET/CT was associated with a higher specificity for detecting EPE [19]. Besides local staging information, PSMA PET/CT enables calculation of quantitative parameters, which might also further improve EPE risk prediction [20]. Based on these favourable results regarding PSMA PET/CT local staging ability, it is conceivable that integrating PSMA PET staging information could further improve EPE risk prediction.

This study aimed to develop and externally validate a side-specific prediction model for EPE incorporating PSMA PET/CT-derived variables alongside MRI and clinical parameters, and to evaluate its performance, in terms of discrimination, calibration, and net benefit, in comparison with an existing MRI-based model (Soeterik).

## 2 Patients and methods

### 2.1 Patient population and study data

The patient population was derived from a previously published multicentre cohort of men who underwent RP, with or without pelvic lymph node dissection, at seven tertiary referral centres and who were preoperatively staged with PSMA PET/CT between May 2016, when PSMA PET/CT became available for preoperative staging, and June 2023 [21] (Supplementary File 1). Patients were required to be treatment naive at the time of imaging. The indication for preoperative PSMA PET/CT was determined by the local multidisciplinary team, reflecting real-world practice. Because PSMA PET/CT was introduced at different time points across centres, the contributing period varied per centre. At two centres, patients enrolled in the prospective PSMA Select trial [22] were managed within that trial and were not included in the present retrospective cohort; apart from this, consecutive patients were included at all centres.

Of the 605 patients in this cohort, 48 were excluded because no presurgical MRI was available, leaving 557 patients. The cohort was subsequently divided by centre into a validation cohort (centre 1; *n* = 146) and a development cohort (remaining centres; *n* = 411). Because the prediction model is side specific, all analyses were performed at the level of the prostatic lobe. Lobes with missing data for any of the model variables (Standardized Uptake Value max (SUV_max_), PSMA_total_, PSAD, or MRI T-stage) were excluded at this stage. This resulted in 801 lobes from 401 patients in the development cohort and 287 lobes from 144 patients in the validation cohort. The full selection process, including the number of patients excluded at each step, is now presented in a flow diagram (Supplementary File 2A). The institutional review boards approved the study prior to initiating data collection (AW24.048/W18.055). Written informed consent was waived because of the retrospective nature of the study.

### 2.2 Development and validation cohort

For nomogram development, data from six international tertiary centres (The Netherlands excluding St Antonius Hospital, Germany, Italy, USA, and Hong Kong) were used, including 411 patients, comprising 822 prostate lobes, of which 21 were excluded because of missing values. The validation cohort included 146 patients from St. Antonius Hospital (The Netherlands), comprising 292 lobes, of which five were excluded because of missing values. Supplementary File 2B presents the flowcharts illustrating the inclusion of both the development and validation cohorts.

### 2.3 MRI acquisition and interpretation

MRI scans were evaluated by experienced uroradiologists following Prostate Imaging-Reporting and Data System version 2.1 criteria [23]. Both multiparametric MRI (mpMRI; T1-weighted [T1W], T2-weighted [T2W], diffusion-weighted imaging [DWI], and dynamic contrast-enhanced [DCE] sequences) and biparametric MRI (bpMRI; T1W, T2W and DWI, excluding DCE) were accepted. Radiology reports were analysed to extract the presence of EPE and seminal vesicle invasion (SVI) separately for each prostate side.

### 2.4 PSMA PET/CT acquisition and interpretation

All PSMA PET/CT scans were performed according to local protocols, as described previously [21]. External scans were accepted and were re-evaluated by the local team. PET/CT images were acquired from the midthigh to the skull base and combined with either low-dose or diagnostic CT for anatomical correlation. All scans were reported by an experienced nuclear medicine physician or trained research fellow under the direct supervision of a nuclear medicine staff physician, both blinded to MRI results. The radiotracers administered were [^68^Ga]Ga-PSMA-11, [^18^F]PSMA-1007, [^18^F]DCF-PyL, and [^18^F]JK-PSMA-7. Administered activities ranged from weight-based dosing to fixed doses (∼1.5–2.0 MBq/kg for [^68^Ga]Ga-PSMA-11 and 250 MBq or weight-adjusted dosing for [^18^F]PSMA-1007). Image acquisition was performed 60–90 min after tracer injection. Patients were hydrated according to local practice and were asked to void their bladder prior to imaging (Supplementary File 3). Image acquisition was performed according to European Association of Nuclear Medicine/Society of Nuclear Medicine and Molecular Imaging guidelines [24]. Local staging was done in accordance with the Prostate Cancer Molecular Imaging Standardized Criteria (PROMISE) version 2 molecular imaging (mi) tumour-node-metastasis staging system [25]. EPE (miT3a) was defined as a tumour extending beyond the prostate contour on CT and/or morphological signs such as an angulated prostate contour or obliterated rectoprostatic angle. SVI (miT3b) was defined as PET tumour activity extending into the seminal vesicle and/or separate focal activity in the seminal vesicle [26].

### 2.5 Histopathological evaluation

Histopathological assessment of RP specimens, including whole-gland and side-specific staging, was used as the reference standard. Tissue processing followed European Association of Urology–ISUP guidelines, using either conventional sectioning or whole-mount techniques per institutional protocol [27]. Local uropathologists performed evaluation and grading in accordance with ISUP recommendations [28]. EPE (pT3a) was defined as tumour extension beyond the prostate capsule, including invasion into periprostatic fat, posterolateral connective tissue, the neurovascular bundle, or microscopic bladder neck involvement. SVI (pT3b) was defined as tumour infiltration of the seminal vesicle muscle wall [29]. The presence and laterality (left, right, or bilateral) of EPE and SVI were routinely recorded and extracted from pathology reports.

### 2.6 Definitions of variables and predicted outcome

Preoperative parameters that were collected included age, initial PSA (iPSA), clinical tumour stage assessed by digital rectal examination, MRI tumour (T)-stage, and biopsy ISUP GG. Furthermore, PSMA T-stage and PSMA parameters, including PSMA_volume_, SUV_max_, and PSMA_total_, were collected. For the calculation of the PSAD, the iPSA and prostate volume, measured by MRI, were used. PSMA quantitative parameters were defined as follows: SUV_max_ was defined as the maximum standardised uptake value, and total PSMA uptake (PSMA_total_) was calculated as the product of SUV_mean_ and the volume of the region of interest. PSMA parameters were assessed by manual delineation of PSMA-expressing tumour lesions within the prostate, using an SUV threshold of ≥4 to define the volume of interest [30]. Body weight SUV normalisation was used across all centres. The primary outcome was side-specific EPE, determined on RP pathology and defined as a pathological tumour stage of pT3a or higher (ie, pT3a or pT3b) in the corresponding lobe.

### 2.7 Predictors and model building

Because each patient could contribute two prostate lobes, observations were clustered within patients. Nonlinearity of continuous variables was evaluated using restricted cubic splines with four knots. Variable selection was performed by backward elimination under the generalised estimating equations (GEE) specification, using the quasi-likelihood information criterion (QIC), the analogue of Akaike Information Criterion (AIC) for models estimated by GEE. At each step, the term whose removal yielded the largest reduction in QIC was eliminated, until no further reduction was achieved. The full model contained all parameters of the previously developed MRI-based nomogram [13]: PSAD, highest ipsilateral ISUP GG, and side-specific stage assessed by MRI. Additionally, PSMA PET/CT variables were included: PSMA T-stage, side-specific SUV_max_, and PSMA_total._ These PSMA PET parameters were selected as they have been repeatedly shown to be associated with PCa outcomes [20], [21], [31]. The retained specification was subsequently refitted using GEE, with the patient as the clustering unit, an exchangeable working correlation structure, and robust sandwich standard errors. Clusters contained at most two observations, and the estimated within-patient working correlation was ρ = 0.223 (Standard error (SE) 0.057). All coefficients, confidence intervals (CIs), and *p* values reported in this study are derived from this GEE model. A comparison with the identical specification fitted as a standard logistic regression without clustering correction is provided in Supplementary File 4.

Coefficient re-estimation was performed, rather than a fixed-coefficient extension of the original model. Categorical covariates were modelled as follows. Biopsy ISUP GG was categorised as 1 (including GGs 0–1, combined because of low numbers), 2, 3, 4, and 5, with GG 1 as the reference. MRI T-stage was categorised as organ-confined with no lesion (NL), organ-confined with a lesion (OC), and EPE including SVI (EPE/SVI combined), with NL as the reference. PSMA T-stage was categorised analogously as NL, OC, and EPE, with NL as the reference. PSAD was modelled as a continuous variable (per [1/0.1] unit) and PSMA_total_ per 100 units.

### 2.8 Model performance, external validation, and clinical usefulness

Evaluation and performance assessment of the prediction model were conducted in accordance with recognised guidelines for prediction model research, including TRIPOD [32]. The sample size was guided by the number of outcome events. The events per variable (EPV) in the development cohort was 31, which is in line with accepted recommendations [33]. The EPV in the validation cohort was seven. To quantify the incremental value of PSMA PET/CT beyond clinical and MRI parameters, the final model was compared with a nested model containing the clinical and MRI predictors only. The added block comprised the PSMA-derived variables retained after backward elimination, namely PSMA_total_ and PSMA T-stage. Because the models were estimated using generalised estimating equations, which are based on quasi-likelihood and do not yield a conventional likelihood, the likelihood ratio test was replaced by its GEE analogue, a Wald test on the added PSMA terms. This comparison was performed in the development cohort.

Model discrimination, defined as the ability of the model to distinguish a prostate lobe with EPE from a lobe without EPE, was quantified using the area under the receiver operating characteristic curve (AUC) [34]. The AUC ranges between 0.5 and 1, with higher values indicating better discrimination between patients with the endpoint and without the endpoint [35]. Model calibration, reflecting the agreement between predicted probabilities and observed outcomes, was evaluated using calibration-in-the-large (systematic overprediction or underprediction) and calibration slope [34]. The net benefit per risk threshold was determined using decision-curve analysis (DCA). Net benefit, assessed across risk thresholds of 0–50%, reflects the number of true positives gained by using the model, after adjusting for false positives at each threshold [36]. Therefore, the 0–50% range reflects probability levels at which imaging-derived information meaningfully contributes to postimaging risk stratification, before definitive therapeutic decisions, such as surgical planning, are made by the urologist or multidisciplinary team. The original MRI-based nomogram (Soeterik model 2) was applied using its published coefficients, without refitting, in both the development and the validation cohort [13]. Discrimination, calibration, and net benefit were assessed for both nomograms in each cohort, allowing direct comparison of performance within the same populations. DeLong test was used to assess statistically significant differences in AUCs between models. Effect modification by PSMA tracer type was assessed by adding interaction terms between tracer type and all covariates and testing the full interaction block with a Wald test. For statistical analysis, R version 4.4.0 (R Foundation for Statistical Computing, Vienna, Austria) was used.

Missing data were present in 26 of the 1114 sides (2.3%) and were handled using complete-case analysis. Missingness was distributed across several variables (PSMA_total_, *n* = 10; MRI T-stage, *n* = 7; PSAD, *n* = 6; SUV_max_, *n* = 5; surgical T3a, *n* = 4; PSMA T-stage, *n* = 2) and was predominantly isolated, with 20 of the 26 sides missing only a single variable. Excluded and included sides were compared on all observed variables. Excluded sides had higher PSMA_total_ (118.7 vs 26.8, *p* < 0.001) and differed in centre distribution (*p* < 0.001; range 0–17.5% per centre), whereas PSAD, SUV_max_, iPSA, biopsy ISUP grade, MRI T-stage and side did not differ significantly. As missingness was associated with observed variables (notably centre and PSMA_total_) rather than occurring completely at random, data were considered missing at random conditional on observed covariates. Given the low proportion of missing data, complete-case analysis was considered unlikely to introduce meaningful bias; multiple imputation was therefore not deemed necessary. All tests were two sided, with statistical significance defined as *p* < 0.05.

## 3 Results

### 3.1 Baseline characteristics

In total, 545 patients (1088 lobes) met the inclusion criteria, with histopathologically confirmed EPE in 377 lobes (35%). The development cohort included 401 patients, whereas the validation cohort comprised 144 patients (Table 1). The side-specific prevalence of EPE was 308 of the 801 lobes (39%) in the development cohort and 69 of the 287 lobes (24%) in the validation cohort (Supplementary File 5).

**Table 1:** Baseline characteristics at the patient level

	Development cohort (*n* = 401)	Validation cohort (*n* = 144)
Age (yr), median (IQR)	66 (61–71)	68 (64–73)
PSA (ng/ml), median (IQR)	9.6 (6.6–16)	9.5 (5.7–19)
PSA density (ng/ml/ml), median (IQR)	0.20 (0.11–0.37)	0.22 (0.15–0.39)
Clinical T-stage, *n* (%)		
T1a-c	187 (47)	70 (49)
T2a	129 (32)	31 (22)
T2b	23 (5.7)	9 (6.2)
T2c	5 (1.2)	6 (4.2)
T3a-b/T4	48 (12)	21 (15)
Tx	9 (2.2)	7 (4.9)
MRI T-stage, *n* (%)		
T0	11 (2.7)	7 (4.9)
T2a/T2	149 (37)	62 (43)
T2b	26 (6.5)	4 (2.8)
T2c	44 (11)	23 (16)
T3a	128 (32)	37 (26)
T3b	42 (11)	10 (6.9)
T4	1 (0.2)	1 (0.7)
PSMA T-stage, *n* (%)		
miT0	11 (2.7)	13 (9.0)
miT2	247 (62)	110 (76)
miT3a	90 (22)	3 (2.1)
miT3b	43 (11)	17 (12)
miT4	10 (2.5)	1 (0.7)
SUV_max_, median (IQR)	10 (6.6–18)	7.4 (4.6–13)
PSMA_volume_, median (IQR)	5.3 (2.3–11)	2.4 (0.2–6.3)
PSMA_total_, median (IQR)	34 (12–76)	12 (1.0–37)
PSMA radioligand, *n* (%)		
[^68^Ga]Ga-PSMA-11	184 (46)	136 (94)
[^18^F]PSMA-1007	192 (48)	0 (0.0)
[^18^F]DCF-PyL	6 (1.5)	0 (0.0)
[^18^F]JK-PSMA-7	16 (4.0)	6 (4.2)
Unknown	3 (0.7)	2 (1.8)
Biopsy ISUP GG, *n* (%)		
Benign/unknown	3 (0.7)	–
1	18 (4.5)	5 (3.5)
2	104 (26)	32 (22)
3	109 (27)	53 (37)
4	99 (25)	49 (34)
5	68 (17)	5 (3.5)

PSA = prostate-specific antigen; IQR = interquartile range; MRI = magnetic resonance imaging; PSMA = prostate-specific membrane antigen; ISUP = International Society of Urological Pathology; GG = grade group; SUVmax = Maximum Standardized Uptake Value.

### 3.2 Multivariable logistic regression

Nonlinear modelling did not improve model fit for PSAD (*p* = 0.081) or PSMA_total_ (*p* = 0.7), and both variables were therefore included as linear terms. The full multivariable model for predicting surgical EPE consisted of PSAD, biopsy ISUP GG, MRI T-stage, PSMA T-stage, SUV_max_, and PSMA_total_. After backward elimination by minimising QIC (Supplementary File 6), the final model retained PSAD, biopsy ISUP GG, MRI T-stage, PSMA T-stage, and PSMA_total_ (per 100 units; Table 2). QIC of the final model was 853. Collinearity analysis revealed weak to moderate collinearity between all parameters, with variance inflation factor values ranging from 1 to 5 (Supplementary File 7). The updated PSMA-MRI combined nomogram is shown in Fig. 1. The coefficients of the updated PSMA-MRI combined nomogram and original MRI-based nomogram are demonstrated in Supplementary File 8.

**Table 2:** Results of the final GEE model for side-specific EPE, accounting for clustering of prostate sides within patients

	OR (95% CI)	*p* value
PSA density	1.54 (0.94–2.50)	0.084
Biopsy ISUP		
Benign/1	Referent	
2	2.01 (1.27–3.19)	0.003
3	3.82 (2.30–6.35)	<0.001
4	2.50 (1.54–4.08)	<0.001
5	4.05 (2.18–7.53)	<0.001
MRI T-stage		
No lesion	Referent	
OC	1.31 (0.86–1.99)	0.2
EPE	4.11 (2.55–6.60)	<0.001
PSMA T-stage		
No lesion	Referent	
OC	1.67 (1.07–2.61)	0.025
EPE	2.59 (1.49–4.49)	<0.001
PSMA_total_^a^	1.72 (1.33–2.23)	<0.001

GEE = generalised estimating equations; OR = odds ratio; PSA = prostate-specific antigen; CI = confidence interval; MRI = magnetic resonance imaging; OC = organ confined; EPE = extraprostatic extension; PSMA = prostate-specific membrane antigen; ISUP = International Society of Urological Pathology; GG = grade group.

a PSMA_total_ per 100 units.

**Fig. 1:**
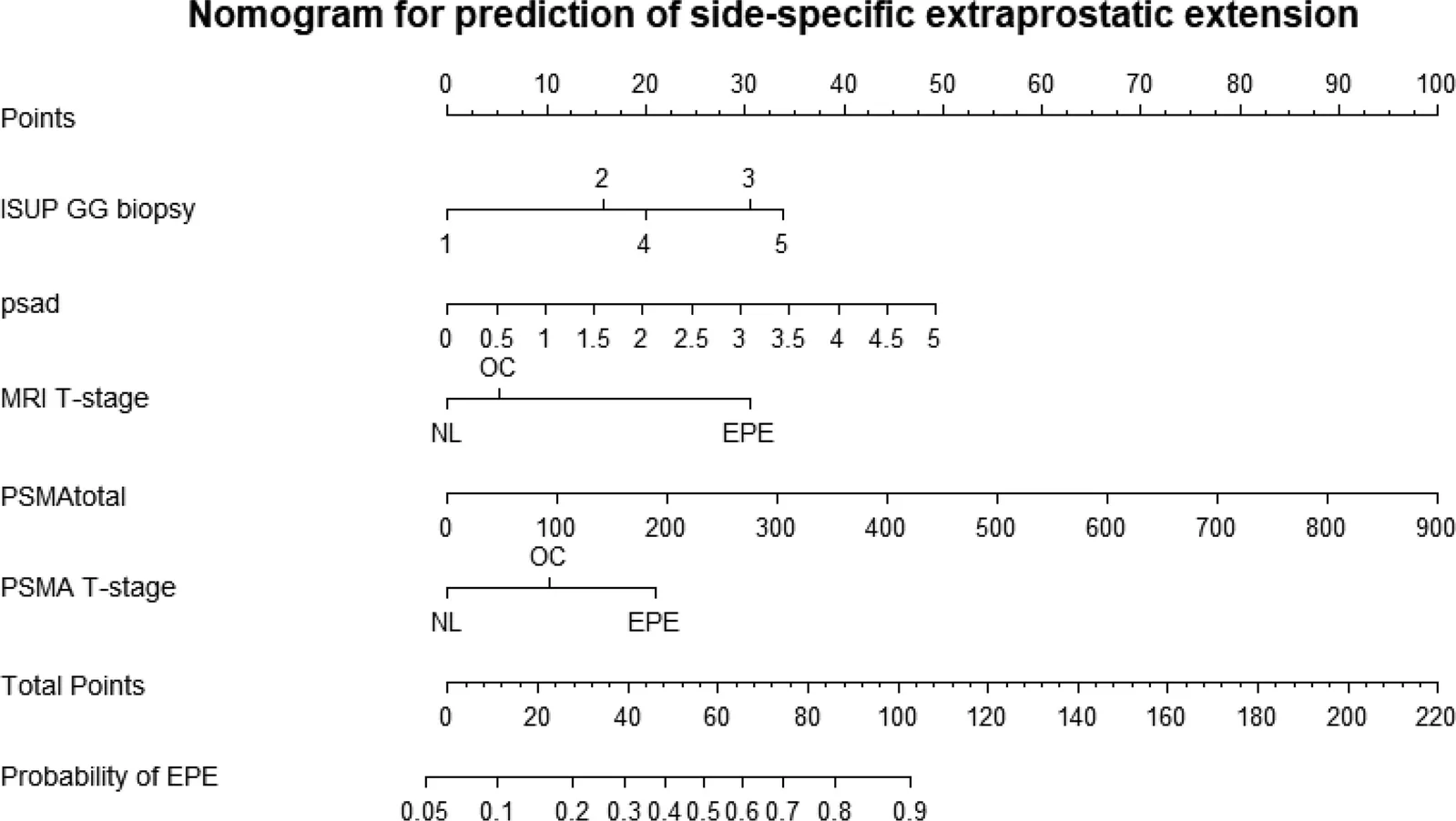
PSMA-MRI nomogram for prediction of EPE. The “points” and “total Points” rows represent the nomogram scoring scale and are not model variables; predictor coefficients are reported in Supplementary File 8. EPE = extraprostatic extension; ISUP = International Society of Urological Pathology; GG = grade group; MRI = magnetic resonance imaging; NL = no lesion; OC = organ confined; PSAD = prostate-specific antigen density; PSMA = prostate-specific membrane antigen.

### 3.3 Model performance of the updated PSMA-MRI combined nomogram in the development cohort and comparison with the original MRI-based nomogram

In the development cohort, adding the PSMA-derived predictors to a model containing the same clinical and MRI predictors, refitted in the present cohort, statistically significantly improved model fit (Wald c^2^ = 32.1, *p* < 0.001), with a modest accompanying gain in discrimination (AUC 0.78 [95% CI 0.75–0.81] to 0.80 [95% CI 0.77–0.83]). Calibration of the previously developed MRI-based nomogram was externally validated in the development cohort using its original published coefficients. It showed an AUC of 0.77 (95% CI 0.73–0.80). Calibration deviated from ideal, with a slope of 0.81 (95% CI 0.68–0.95) and an intercept of 0.24 (95% CI 0.07–0.41), indicating somewhat too extreme predictions together with a systematic underestimation of the observed EPE risk (Fig. 2A). Adding interactions between tracer type and all covariates improved model fit (Wald c^2^ = 23.5, degrees of freedom = 10, *p* = 0.009), driven predominantly by MRI T-stage, with no interaction for PSAD, PSMA_total_, or PSMA T-stage. Because the tracer type was fully confounded with the centre, the interaction terms were not retained in the final model. Model performance per tracer is shown descriptively in Supplementary File 9.

**Fig. 2:**
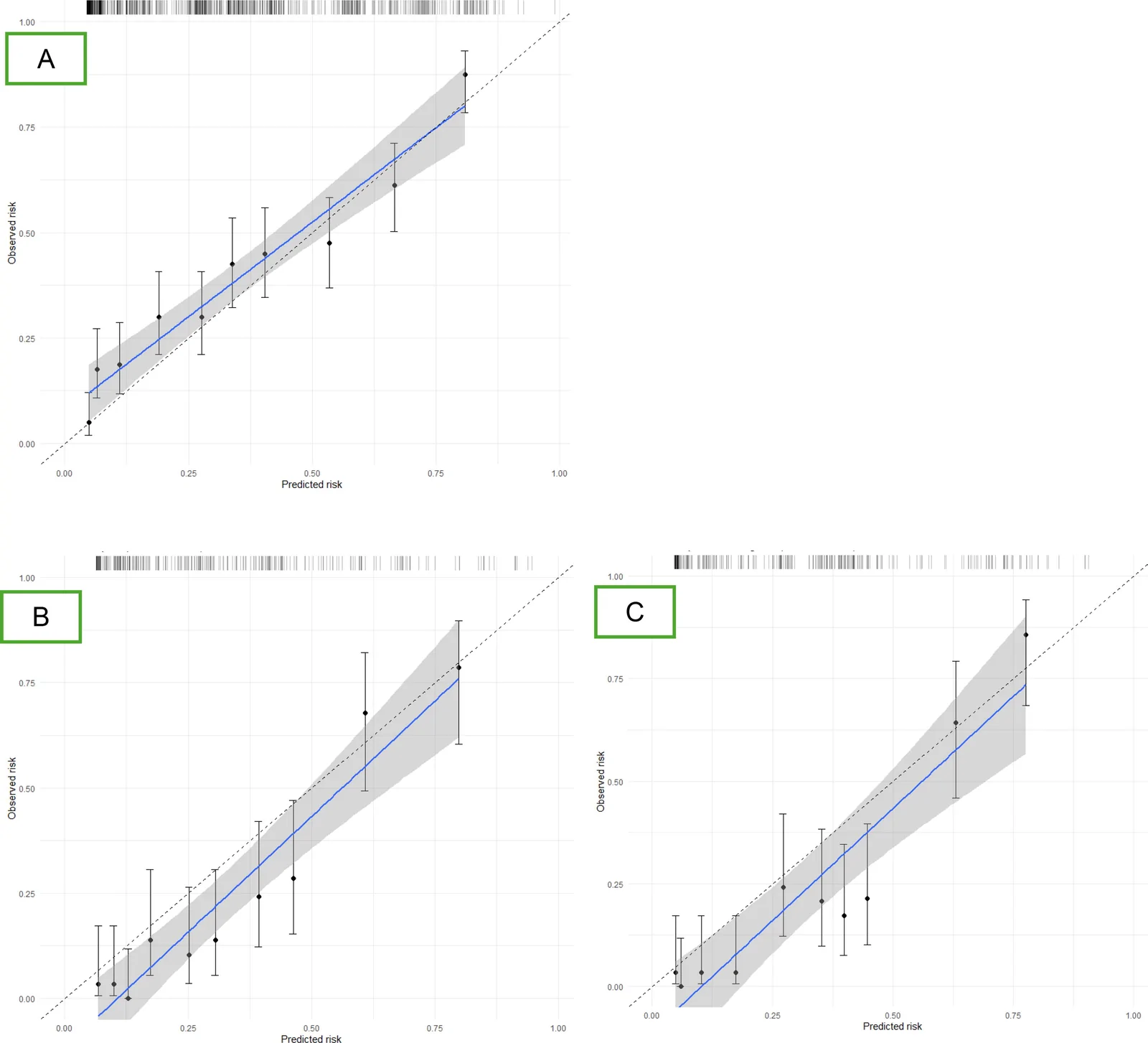
Calibration plot with confidence interval of the original MRI-based nomogram in the development cohort (A). Calibration plots of the updated PSMA-MRI combined nomogram (B) and original MRI-based nomogram (C) in the validation cohort. MRI = magnetic resonance imaging; PSMA = prostate-specific membrane antigen.

On DCA, both nomograms showed superior net benefit compared to the “treat none” and “treat all” strategies for risk thresholds of 10–50%. The updated PSMA-based nomogram showed higher net benefit compared to the MRI-based model for risk thresholds of 20–50% (Fig. 3); an overview per risk threshold is provided in Supplementary File 10.

**Fig. 3:**
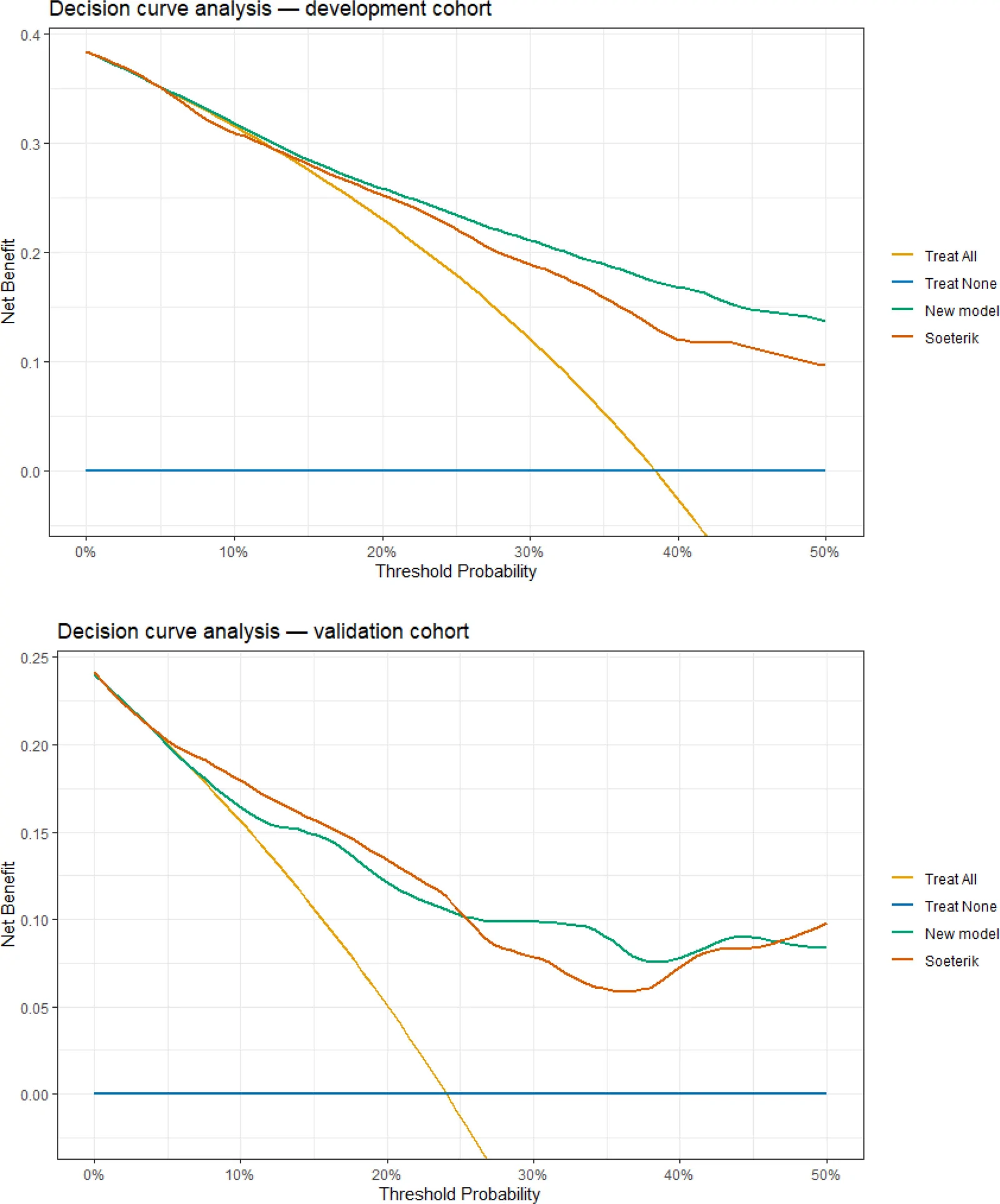
Decision curve analysis of the original MRI-based Soeterik nomogram (red) and updated PSMA-MRI combined nomogram (green) in the validation cohort (bottom) and development cohort (top). MRI = magnetic resonance imaging; PSMA = prostate-specific membrane antigen.

### 3.4 Model performance of the updated PSMA-MRI combined nomogram in the validation cohort and comparison with the original MRI-based nomogram

In the validation cohort, model discrimination in terms of AUC was 0.85 (95% CI 0.80–0.90) for the updated PSMA-based nomogram versus 0.86 (95% CI 0.80–0.91) for the original MRI-based nomogram (*p* = 1.000; Table 3). In terms of calibration in the validation cohort, both tools deviated from ideal calibration to a comparable degree, with a calibration slope of 1.48 (95% CI 1.12–1.89) versus 1.47 (95% CI 1.11–1.89) and a calibration intercept of –0.55 (95% CI –0.86 to –0.25) versus –0.54 (95% CI –0.85 to –0.24) for the updated and the MRI-based nomogram, respectively. The slope above unity indicates that the range of predicted risks was too narrow, whereas the negative intercept reflects a systematic overestimation of the absolute EPE risk (Figs. 2B and C). At a predicted risk threshold of 20%, the updated nomogram identified EPE with a sensitivity of 0.91 (0.82–0.96), a specificity of 0.47 (0.40–0.53), a positive predictive value (PPV) of 0.35 (0.29–0.42), a negative predictive value (NPV) of 0.94 (0.88–0.97), and a false-negative rate of 0.09 (6/69 missed EPE cases). At a 30% threshold, sensitivity was 0.86 (0.75–0.92), specificity 0.67 (0.60–0.73), PPV 0.45 (0.37–0.54), NPV 0.94 (0.89–0.96), and the false-negative rate 0.14 (10/69). Performance across the full range of thresholds is presented in Supplementary File 10.

**Table 3:** Per nomogram and cohort AUC with 95% CI

Nomogram	AUC (95% CI)
Novel PSMA-MRI combined nomogram in development cohort	0.80 (0.77–0.83)
Novel PSMA-MRI combined nomogram in validation cohort	0.85 (0.80–0.91)
Original MRI-based nomogram in development cohort	0.77 (0.73–0.80)
Original MRI-based nomogram in validation cohort	0.86 (0.80–0.91)

AUC = area under the receiver operating characteristic curve; CI = confidence interval; MRI = magnetic resonance imaging; PSMA = prostate-specific membrane antigen.

DCA showed comparable net benefit for both models at lower thresholds, whereas the updated nomogram yielded higher net benefit from ∼20% upwards. At the 30% threshold, net benefit was 0.10 for the updated model and 0.08 for the MRI-based model, both exceeding the “treat all” and “treat none” strategies (Fig. 3).

## 4 Discussion

In this study, our updated nomogram incorporating PSMA PET/CT-derived variables showed improved apparent performance for side-specific EPE compared with an MRI-based model in the development cohort. However, this advantage was not confirmed on external validation, in which the updated model performed comparably to the existing MRI-based model. These findings indicate that incorporating PSMA PET/CT-derived variables into a dedicated prediction model offers limited and possibly context-dependent incremental value over clinical and MRI predictors, rather than a consistent improvement in predictive performance.

Although prior studies have suggested added value of PSMA PET/CT for local staging [17], [18], [19], both models performed similarly in external validation. This may reflect the contribution of established predictors in the original model, such as PSAD and biopsy-derived ISUP GG, potentially attenuating the added value of PSMA PET/CT. Supporting this, PSAD was a statistically significant predictor of EPE in the MRI-based model but not in the multivariable analysis of the updated model. Additionally, both models overestimated side-specific EPE risk, possibly because of a lower prevalence of pathological EPE in the validation cohort. Our study findings align with Tillu et al [37] who recently developed an updated nomogram incorporating ^18^F-DCFPyL PSMA PET/CT alongside established clinical and MRI parameters, showing that the model outperformed their institutional MRI-only nomogram. The added value of our study lies in external validation, providing a more nuanced assessment of the incremental benefit of PSMA PET/CT.

The limited benefit of PSMA PET/CT may also relate to the lack of standardised criteria for local staging. Although frameworks such as PROMISE version 2 have been introduced, detailed descriptors for local extension remain limited, which may increase interobserver variability and contribute to heterogeneity across centres [25], [38] and limit PSMA PET/CT consistency in clinical practice. This is reflected by hospital-level variation in reporting and by inconsistent findings across studies [17], [18], [19], [39], [40]. In contrast, MRI-based EPE assessment relies on more standardised criteria, potentially leading to more consistent staging across institutions [41], [42].

Further improvements in EPE prediction may be achieved through refinement of PSMA PET techniques and exploration of quantitative biomarkers. For instance, Laudicella et al [20] demonstrated that quantitative PSMA PET parameters have similar predictive value to mpMRI-derived longest capsular contact for assessing EPE. Additionally, higher SUV_max_ has been associated with shorter biochemical recurrence-free survival [43], [44]. Quantitative PSMA PET metrics are also linked to surgical ISUP GG and upgrading [21], [45]. Our results support their integration, as reflected by the inclusion of PSMA_total_ in the updated model.

The clinical value of a side-specific EPE model lies primarily in identifying lobes at sufficiently low risk for nerve-sparing surgery to be offered safely. At a predicted risk of 20%, 108 of the 287 lobes (38%) in the validation cohort fell below the threshold, of which six (6%) harboured EPE. Despite an observed EPE prevalence of 24%, the model therefore distinguishes a substantial proportion of lobes at low risk. Raising the threshold to 30% would increase the proportion, falling below the threshold from 38% to 54% at an unchanged residual risk of 6%, while the number of missed EPE cases increases from six to ten. Conversely, of the lobes at or above the threshold, 116 of the 179 (65%) at 20% and 72 of the 131 (55%) at 30% had no EPE and would potentially be denied nerve-sparing surgery unnecessarily. The appropriate threshold therefore depends on the weight attached to functional outcomes (ie, erectile function) relative to the risk of a positive surgical margin, and on local availability of intraoperative frozen-section analysis. Performance characteristics across the full range of clinically plausible thresholds are provided in Supplementary File 10. Beyond guiding nerve-sparing decisions, side-specific EPE risk prediction could in principle be used to target intraoperative frozen-section analysis to patients in whom the decision is least certain, rather than applying it universally. Establishing at which predicted risk this is worthwhile would require data on how often frozen-section analysis alters management and prevents a positive surgical margin, which the present cohort does not allow. This application therefore warrants prospective evaluation.

Although the potential of PSMA PET/CT for improving EPE prediction is promising, it is important to acknowledge its current limitations. The relatively low spatial resolution of the CT component may inherently restrict local tumour assessment. Another limitation of PSMA ligands, particularly [^68^Ga]Ga-PSMA-11 and [^18^F]DCFPyL, is their high renal excretion, which may impair the evaluation of lesions in the prostate, especially those located near the bladder neck [46]. Finally, prior studies demonstrated that forced diuresis, use of diuretics, and hydration to clear urine tracer activity, especially in the bladder, reduce artefacts and improve detection of pelvic/local PCa [47], [48]. Improving PSMA PET/CT for EPE assessment will require better spatial resolution, reduced urinary tracer activity, and more standardised imaging protocols. PSMA PET/MRI may overcome limitations of PSMA PET/CT by combining molecular and high soft-tissue contrast imaging. It has shown higher sensitivity for EPE detection than mpMRI and PSMA PET/CT, although not always statistically significant [49]. Its limited availability may restrict broader implementation.

Our study has several limitations, several of which have direct implications for the interpretation and generalisability of our findings. First, the relatively small external validation cohort may limit the robustness of performance estimates. Second, heterogeneity in imaging protocols, radioligands, and scanners across centres may have affected generalisability and introduced variability in quantitative measurements. Although the use of different PSMA radioligands could influence the applicability of the predefined SUV_max_ ≥4 threshold, prior analyses demonstrated comparable SUV_max_ distributions per ISUP GG and similar diagnostic performance across tracers, supporting its use [21]. In addition, patient selection for PSMA PET/CT was not standardised and evolved over time, introducing potential selection bias. Relatedly, the development cohort had a higher EPE prevalence than the validation cohort (39% vs 24%), reflecting a difference in case mix: the development cohort comprised more high-risk patients (ISUP 5: 17% vs 3.5%; MRI ≥T3a: 43% vs 33%), most likely driven by differences in referral and surgical-selection patterns across centres. As models developed in higher-prevalence populations tend to overestimate absolute risk in lower-prevalence settings, this case mix difference likely limits the transportability of the predicted probabilities at external validation. This is supported by the negative calibration intercept observed in our validation cohort, reflecting systematic overestimation of absolute EPE risk. Imaging was interpreted locally without central review, which may particularly affect qualitative assessments such as EPE because of interreader variability. The absence of central histopathological review may also have influenced our results. Although reporting followed international uropathology guidelines, interobserver variability in EPE assessment is well documented, with agreement among expert uropathologists being high for unequivocal cases but substantially lower for equivocal ones [50]. Because EPE was our outcome, such variability introduces potential outcome misclassification, and the model may overrepresent or underestimate risk in settings where systematically stricter or less strict EPE criteria are applied. A further limitation is the complete-case analysis because missing PSMA PET parameters could not be retrieved retrospectively. Although missingness was related to centre rather than to patient or tumour characteristics, residual bias and reduced generalisability cannot be excluded. Another limitation is the use of stepwise variable selection, which is known to increase the risk of overfitting and optimism. Internal validation with correction for overfitting was not performed, and the estimated coefficients are therefore to some extent sample specific. In addition, EPE was modelled as a dichotomous outcome, whereas focal capsular penetration carries different oncological implications than established EPE; a more graduated prediction would require larger cohorts with standardised grading of EPE extent. Finally, both mpMRI and bpMRI were used, and the proportion per cohort was not recorded. Although prior studies have reported comparable performance for EPE detection [51], our coefficients therefore reflect a mixture of both protocols, and it remains uncertain whether they transport equally well to populations imaged exclusively with either modality.

For further research, a larger validation cohort, prospectively harmonised cohorts, standardised acquisition protocols and centralised image review will be essential to further refine and optimise the combined nomogram.

## 5 Conclusions

Adding PSMA PET/CT-derived variables improved side-specific EPE prediction at model development, but we were unable to demonstrate improved discrimination on external validation. Therefore PSMA PET/CT should not be performed for the sole purpose of side-specific EPE assessment. Because model performance depends on the case mix of the population studied, prospective evaluation in a cohort with uniform imaging indications is needed before this model can be used to guide nerve-sparing decisions.

  ***Author contributions***: Vera Sweere had full access to all the data in the study and takes responsibility for the integrity of the data and the accuracy of the data analysis.

*Study concept and design: Vera Sweere, Timo F.W. Soeterik, Matthijs J.V. Scheltema.*

*Acquisition of data: Vera Sweere, Alexandra Bruins Slot, Joris G. Heetman, Rick Hermsen, Lieke Wever, Maarten Vinken, Clint D. Bahler, Mark Tann, Claudia Kesch, Tugce Telli, Peter Ka-Fung Chiu, Fabio Zattoni, Laura Evangelista, Timo F.W. Soeterik.*

*Analysis and interpretation of data: Vera Sweere.*

*Drafting of the manuscript: Vera Sweere, Timo F.W. Soeterik.*

*Critical revision of the manuscript for important intellectual content: Jules Lavalaye, Maarten Vinken, Clinton D. Bahler, Mark Tann, Claudia Kesch, Tugce Telli, Wolfgang P. Fendler, Peter Ka-Fung Chiu, Kwan Kit Wu, Fabio Zattoni, Laura Evangelista, Francesco Ceci, Marcin Miszcyzyk, Pawel Rajwa, Akihiro Matsukawa, Tamás Fazekas, Francesco Barletta, Alberto Briganti, Francesco Montosori, Giorgio Gandaglia, Jean-Paul A. van Basten, Harm H.E. van Melick, Roderick C.N. van den Bergh, Giancardlo Marra, Matthijs J.V. Scheltema, Timo F.W. Soeterik.*

*Statistical analysis: Vera Sweere.*

*Obtaining funding: N/A.*

*Administrative, technical, or material support: Alexandra Bruins Slot, Joris heetman, Lieke Wever, Rick Hermsen.*

*Supervision: Timo F.W. Soeterik, Matthijs J.V. Scheltema.*

*Other (specify): N/A.*

  ***Financial disclosures*:** Vera Sweere certifies that all conflicts of interest, including specific financial interests and relationships and affiliations relevant to the subject matter or materials discussed in the manuscript (eg, employment/affiliation, grants or funding, consultancies, honoraria, stock ownership or options, expert testimony, royalties, or patents filed, received, or pending), are the following: Marcin Miszczyk was supported by the European Urological Scholarship Programme of the European Association of Urology. Paweł Rajwa was supported by NAWA–Polish National Agency for Academic Exchange in cooperation with the Medical Research Agency under the Walczak Programme (grant number BPN/WAL/2023/1/00016).

Rick Hermsen received consultancy fees from ABX advance biochemical compounds GmbH and Blue Earth Diagnostics. Wolfgang P. Fendler reports fees from SOFIE Bioscience (research funding), Janssen (consultant and speaker), Perceptive (consultant and image review), Bayer (consultant, speaker, and research funding), Novartis (speaker and consultant), Telix (speaker), GE Healthcare (speaker and consultant), Eczacıbaşı Monrol (speaker), ABX (speaker), Amgen (speaker), Urotrials (speaker), Lilly (consultant), and AstraZeneca (research funding) outside of the submitted work. The remaining authors have nothing to disclose.

  ***Funding/Support and role of the sponsor*:** This work was supported by Cure for Cancer. The funder had no role in the study.

  ***Acknowledgements*:** The authors thank the other members of the European Association of Urology-Young Academic Urologists (EAU-YAU) Prostate Cancer Working Party for their valuable contributions. The scientific *guarantor* of this publication is T.W.F. Soeterik.

  ***Statistics and biometry*:** *N*o complex statistical methods were necessary for this paper.

  ***Informed consent*:** The requirement to obtain informed consent was waived because of the retrospective nature of the study.

  ***Ethics approval*:** The Medical Research Ethics Committees United has reviewed the study protocol and waived the need for approval (AW24.048/W18.055). Institutional review board approval was obtained.

## Appendix A Supplementary data

Supplementary Data 1 Extended methods, supplementary analyses, and additional results not included in the main text.
