# Supplementary material for: Development and External Validation of an Updated Nomogram Including Both MRI and Local PSMA PET/CT Information for the Prediction of Side-specific Extraprostatic Extension in Patients Treated with Radical Prostatectomy
Source: Eur Urol Open Sci. 2026 Oct 29;94:52–63. doi: 10.1016/j.euros.2026.10.003 (PMC13743373; doi:10.1016/j.euros.2026.10.003)
Supplement: Supplementary file 1 [file mmc1.docx]

**Supplementals**

Supplementary file 1 – number of patients from each participating center

| Hospital | Country | Development cohort  (n = 411) | Validation cohort  (n = 146) |
| --- | --- | --- | --- |
| St Antonius Hospital | The Netherlands | 0 | 146 |
| Canisius Wilhelmina Hospital | The Netherlands | 213 | 0 |
| University Hospital Essen | Germany | 41 | 0 |
| Indiana University Medical Center | United States of America | 85 | 0 |
| The Chinese University of Hong Kong | China | 37 | 0 |
| University Hospital of Padova | Italy | 19 | 0 |
| IEO European Institute of Oncology | Italy | 16 | 0 |

Supplementary file 2 – Flowcharts demonstrating inclusion and exclusion at the patient and lobe levels

Supplementary file 2A – flowchart showing patient inclusion and exclusion

Supplementary file 2B – Flowchart demonstrating inclusion and exclusion for the development cohort (upper panel) and validation cohort (lower panel)

Supplementary file 3 – PSMA PET/CT protocol per center

**Table 1. Local PSMA PET protocols**

| **Hospital 1** |  |
| --- | --- |
| **Radioligand preparation** | The [^68^Ga]Ga-PSMA-11 was prepared using a GMP (GoodManufacturing Process) grade 68Ge/68Ga generator (Eckert& Ziegler Strahlen, Berlin, Germany) and a semiautomated synthesis module (ITG, Munich, Germany). Each synthesis was performed according to manufacturer instructions using PSMA-11 ligand (ABX, Radeberg, Germany). |
| **Acquisition and Image reconstruction** | Images were acquired from the skull vertex to the midthigh using a Biograph CT40 scanner or a Gemini TF64 slice scanner (Philips, Best, The Netherlands). Intra-venous 1.5 MBq/kg 68Ga-PSMA-11 was administered followed by 500 ml saline. PET images were acquired 60minutes after injection. Low dose CT was done directly after PET. Images were acquired according to EANM (European Association of Nuclear Medicine) criteria.  Reconstruction and post-filtering: PET reconstructions were made using the scanner’s default Ordered Subset Expectation Maximization (OSEM) reconstruction algorithm with 33 subsets, 3 iterations, matrix size of 144×144, and voxels of 4×4×4 mm. No Gaussian filter was applied. The reconstruction was corrected for geometrical response and detector efficiency (normalization), random coincidences, scatter and attenuation. |
| **Nuclear physician experience** | >5 years and >500 scans |
| **Accreditation** | EARL 1 |

| **Hospital 2** |  |
| --- | --- |
| **Radioligand preparation** | [^18^F]PSMA-1007 was synthesized at the Radboud University  Medical Center translational medicine cyclotron facility.  Reagent kit and PSMA-1007 precursor were obtained from  ABX (Radeberg, Germany). |
| **Acquisition and Image reconstruction** | Images were obtained from skullbase to mid-thigh. PET/CT scans were performed on a Philips Gemini 64TF (Eindhoven, The Netherlands) or a Siemens Biograph Vision 450 (Erlangen, Germany). An fixed dose of approximately 250 MBq of [^18^F]PSMA-1007 was administered intravenously, acquisition started approximately 90 min post injection.  Patients were hyperhydrated and voided before the scan.  Reconstruction and post-filtering: Iterative+TOF with 2 iterations, Gaussian filter, FWHM 7.0mm, image size 220. Voxel size is 5 mm. |
| **Nuclear physician experience** | >5 years and >500 scans |
| **Accreditation** | EARL, in this paper EARL 1 reconstructions were used for measurements |

| **Hospital 3** |  |
| --- | --- |
| **Radioligand preparation** | [^68^Ga]Ga-PSMA-11 and [^18^F]PSMA-1007 were employed for PET imaging. Tracer precursors (PSMA-11 and PSMA-1007) were obtained from ABX advanced biochemical compounds (ABX GmbH). [^18^F]PSMA-1007 and [^68^Ga]Ga-PSMA-11 were synthesized on site using a kit-based approach on automated platforms with comprehensive pH, radiochemical, chemical, and radionuclide purity control tests. Intravenous administration was body-weight–dependent (2 MBq/kg of body weight). |
| **Acquisition and Image reconstruction** | Image acquisition (from the vertex to thigh) started 60 minutes after injection. Patients were asked to void their bladder before imaging. PET/CT was used to acquire coregistrated images. Either low-dose or full-dose CT acquisitions were obtained directly before PET acquisition using a Biograph mCT (Siemens Healthineers, Knoxville, TN, United States). PET emission data were attenuation corrected by help of the CT data and iteratively reconstructed with time-of-flight information and point-spread function correction (HD PET). PET/MRI examination was performed with an integrated 3.0-T Biograph mMR scanner (Siemens Healthineers), and simultaneous PET and 3D Dixon-volumetric interpolated breath-hold examination (VIBE) sequences for MRI-based scatter correction were performed, followed by a standardized whole-body MRI protocol.  Voxel size, reconstruction and post-filtering: For both [^18^F]PSMA-1007 and [^68^Ga]Ga-PSMA-11, attenuation corrected PET images were reconstructed iteratively using three dimensional ordinary Poisson ordered subset expectation maximization algorithm with combined time-of-flight and point-spread function modelling with 3 iterations and 21 subsets. Images were reconstructed into a 200×200 tranverse matrix and a voxel size of 3.3×3.3×3.0 mm. Standard corrections for scatter, randoms, and normalization were applied as well as an isotropic post-reconstruction 4-mm Gaussian filter. |
| **Nuclear physician experience** | >5 years and >1000 scans |
| **Accreditation** | EARL 1 and 2 |

| ***Hospital 4*** |  |
| --- | --- |
| **Radioligand preparation** | The ^68^Ga-PSMA-11 was prepared from the ^68^Ga eluate of either an ITG Isotope Technologies Garching (Germany) GMP^68^Ge/^68^Ga generator (1680 MBq) or an IRE (Fleurus, Belgium) Elit GalliEo ^68^Ge/^68^Ga generator (1850 MBq), 10-µg GMP PSMA-11 (ABX, Radeberg, Germany), and ultrapure sodium acetate buffer using a manual synthesis box (ITG iQS Fluidic Labeling Module). After heating for 5-minutes at 95°C the ^68^Ga-PSMA-11 product was trapped on a conditioned Waters C18 SepPak Light solid phase extraction cartridge, recovered by elution with 1-mL 50% ethanol in saline, diluted to 5% ethanol with sodium chloride for injection, and filtered through a sterile disposable 0.2µm PVDF filter unit before quality control testing and dose release. |
| **Acquisition and Image reconstruction** | Immediately before the PET acquisition a computed tomography (CT) scan of the pelvis was acquired for anatomic correlation and attenuation correction (120 kV peak, 330ms exposure time, 658 mA tube current, 0.98 x 0.98 x 1.00mm voxels, 500mm field of view) using a soft tissue kernel (Br38). Patients then had a 55-minute dynamic PET scan acquired in list-mode, centered over the pelvis, after administration of a nominal 185 MBq dose of ^68^Ga-PSMA-11 (i.v) followed by 10-mL saline flush. Images were acquired from the time of administration using either a Siemens Biograph mCT or a Siemens Biograph Vision 600 Edge scanner (Siemens Healthineers, Knoxville, USA). PET images were reconstructed with a 3D ordered-subsets expectation maximization (OSEM) algorithm with point-spread function (PSF) and time of flight (TOF) [3i5s, 3.5mm FWHM spatial resolution, 210ps temporal resolution, 1.42 x 1.42 x 3.0mm voxels, 5mm Gaussian smoothing]. The images were corrected for decay, attenuation, scatter, dead time, random coincidences, and were detector-normalized. Image analysis for this project employed a static reconstruction of the PET data from 40-55-minutes post-injection. Unless contraindicated, a contrast-enhanced CT image was also collected at the end of the PET acquisition. |
| **Nuclear physician experience** | >5 years and >500 scans |
| **Accreditation** | System testing meets requirements of ACR accreditation for whole body PET systems. |

| **Hospital 5** |  |
| --- | --- |
| **Radioligand preparation** | The [^68^Ga]Ga-PSMA-11 was prepared using a GMP (Good Manufacturing Process) grade 68Ge/68Ga generator (Eckert& Ziegler Strahlen, Berlin, Germany) and a semiautomated synthesis module (ITG, Munich, Germany). Each synthesis was performed according to manufacturer instructions using PSMA-11 ligand (ABX, Radeberg, Germany). |
| **Acquisition and Image reconstruction** | Images were acquired from the vertex to the midthigh using a scanner GE 710 (General Electric, USA). Intra-venous 185 MBq [^68^Ga]Ga-PSMA-11 was administered followed by 500 ml saline. PET images were acquired 60 minutes after injection. Low dose CT was done before PET. Images were acquired according to EANM (European Association of Nuclear Medicine) guidelines.  Voxel size: 2.7344x2.7344x3.2700 mm^3^  Reconstruction: VPFXS (OSEM+PSF+TOF) 3 interactions, 18 subsets.  Post-filtering: Filter type standard, filter cutoff/width 6.5 mm |
| **Nuclear physician experience** | >5 years and >500 scans |
| **Accreditation** | No accreditation |

| **Hospital 6** |  |
| --- | --- |
| **Radioligand preparation** | The [^18^F]PSMA-1007 injection was prepared using an automated synthesis module, Synthera® (IBA, Louvain-La-Neuve, Belgium). The single-use reaction cassette (IFP), chemical precursor and reagents are manufactured (ABX, Radeberg, Germany) according to GMP (Good Manufacturing Practice) requirements, intended for manufacture of radiopharmaceutical preparation. The product injection solution meets acceptance criteria compliant with European Pharmacopoeia (Eur. Ph.) standards. |
| **Acquisition and Image reconstruction** | Pre-examination fasting was not required. Injected activity of 18F-PSMA-1007 was calculated according to patient’s weight (6.5-11.0 mCi). After intravenous injection, patients were requested to take a rest in preparation room for a minimum uptake period of 90 minutes before scanning. Whole-body scans were acquired after urination, spanning from base of skull to upper thigh. Patients performed normal breathing with both arms positioned above head. Non-contrast CT was performed (120 kVp, 90 mAs, pitch 0.8 and rotation time 0.5 second) followed by PET acquisition (static bed, 2 minutes per bed). PET images were reconstructed using parameters optimized for small lesion depiction (4 iterations 4 subsets, gaussian filter 5 mm at FWHM, voxel size 1.65 x 1.65 x 1.5 mm, point spread function and time-of-flight options enabled. |
| **Nuclear physician experience** | >5 years and >500 scans |
| **Accreditation** | No accreditation |

| **Hospital 7** |  |
| --- | --- |
| **Radioligand preparation** | [^68^Ga]Ga-PSMA-11 will be synthesized in the radiochemistry laboratory of the Division of Nuclear Medicine of the European Institute of Oncology (IEO), Milan. Gallium-68 will be produced with 68Ge/68Ga generator (GalliaPharm 68Ge/68Ga generator, Eckert & Ziegler, Germany). 68Ga-PSMA-HBEDCC(Glu-NH-CO-NH-Lys-(Ahx)-[[68Ga]Ga(N,N′-bis-[2-hydroxy-5-(carboxyethyl)benzyl]ethylenediamine-N,N′-diacetic-acid]) (68Ga-PSMA-11) will be prepared in a similar procedure as described by Eder et al. and transferred to cassette-based automated synthesis module.[1] The whole procedure was performed in accordance with the Good Manufacturing Practices (GMP). The system provides a final report, and the resulting solution (final product) is subjected to standard quality controls. |
| **Acquisition and Image reconstruction** | In accordance with procedure guidelines all patients will receive intravenously a dose of 2 MBq/Kg ± 0.2 MBq of [^68^Ga]Ga-PSMA-11. Patients will receive intravenously hydration (0.5 L, saline solution) during uptake. No preparation before the procedure is required.  [^68^Ga]Ga-PSMA-11 PET/CT will be performed in accordance with a standard technique.[2] All patients will undergo PET/CT scan in a dedicated tomograph (GE DMI DR). An attenuation-corrected whole-body scan (vertex to mid thighs) 2 min per bed position starting 60-90 min after tracer injection will be acquired. A low-dose CT scan will be performed for attenuation correction of the PET emission data and for anatomical correlation. |
| **Nuclear physician experience** | >5 years and >500 scans |
| **Accreditation** | EARL 1 |

Supplementary file 4:
Comparison of the final model fitted as a standard logistic regression, ignoring the paired lobe structure, and as a generalized estimating equation (GEE) with the patient as clustering unit, an exchangeable working correlation structure and robust sandwich standard errors. Odds ratios were closely comparable between the two approaches. Most estimates were modestly attenuated under GEE, with the largest relative difference in log-odds being 13.7% (ISUP grade group 5, OR 5.06 versus 4.05); three estimates were marginally higher under GEE (maximum increase 7.2%). Robust standard errors were close to their model-based counterparts (ratio 0.88–1.04). No covariate changed direction, rank order or statistical significance. OR = odds ratio; SE = standard error.

|  | **PSMA-MRI model (GLM)** | **PSMA-MRI model (GEE)** |
| --- | --- | --- |
| **Intercept** | -2.723 | -2.646 |
| **PSA density** | 0.471 | 0.430 |
| **MRI**  No lesion  Organ-confined  EPE | 0.250  1.461 | 0.267  1.413 |
| **ISUP Grade**  Benign/1  2  3  4  5 | 0.753  1.463  0.959  1.622 | 0.698  1.341  0.918  1.400 |
| **PSMA**  No lesion  Organ-confined  EPE | 0.493  1.003 | 0.512  0.951 |
| **PSMAtotal** | 0.005 | 0.543* |

*PSMAtotal per 100 units

Supplementary file 5 - Baseline per lobe presented separately for development and validation cohort

|  | Development cohort (n = 801 lobes) | | Validation cohort (n = 287 lobes) | |
| --- | --- | --- | --- | --- |
|  | No EPE at histopathology | EPE at histopathology | No EPE at histopathology | EPE at histopathology |
| No. of lobes | 493 | 308 | 218 | 69 |
| PSA density, median (IQR) | 0.176 (0.104-0.295) | 0.244 (0.146-0.416) | 0.224 (0.132-0.362) | 0.221 (0.169-0.532) |
| Clinical stage assessed by MRI, n (%)  No lesion visible  Organ confined (T2)  EPE (T3a)  SVI (T3b-T4) | 225 (45.6) 202 (41.0)  61 (12.4) 5 (1.0) | 53 (17.2)  114 (37.0)  89 (28.9) 52 (16.9) | 93 (42.7)  110 (50.5)  11 (5.0) 4 (1.8) | 6 (8.7)  24 (34.8) 32 (46.4)  7 (10.1) |
| Clinical stage assessed by PSMA, n(%)  No lesion visible  OC (T2)  EPE (T3a)  SVI (T3b-T4) | 175 (35.5)  261 (52.9)  47 (9.5)  10 (2.0) | 36 (11.7)  166 (53.9)  57 (18.5)  49 (15.9) | 73 (33.5) 140 (64.2) 2 (0.9)  3 (1.4) | 11 (15.9)  42 (60.9)  1 (1.4) 15 (21.7) |
| SUV_max_ | 5.26 (3.8-9.3) | 9.6 (5.96-16.5) | 4.68 (3.3-7.4) | 8.47 (4.3-12.3) |
| PSMA_volume_ | 4.52 (1.52-9.36) | 7.18 (3.35-15.6) | 2.1 (0.19-4.90) | 4.30 (0.20-10.8) |
| PSMA_total_ | 27.6 (8.02-63.2) | 46.7 (19.4-116) | 10.4 (0.93-31.2) | 20.7 (1.0-69.2) |
| Biopsy ISUP GG, n (%)  Benign/not biopsied  1  2  3  4  5 | 156 (31.6) 80 (16.2)  112 (22.7) 64 (13.0) 57 (11.6)  24 (4.9) | 31 (10.1)  16 (5.2) 70 (22.7)  79 (25.6) 56 (18.2) 56 (18.2) | 79 (36.2) 25 (11.5)  36 (16.5)  45 (20.6)  32 (14.7) 1 (0.5) | 2 (2.9) 2 (2.9)  14 (20.3)  23 (33.3)  22 (31.9)  6 (8.7) |

Abbreviations: PSA (Prostate Specific Antigen); IQR (interquartile range); MRI (magnetic resonance imaging); EPE (extra prostatic extension); SVI (seminal vesicle involvement); PSMA (Prostate Specific Membrane Antigen); ISUP GG (International Society of Urological Pathology Grade Group)

Supplementary file 6 – Backward elimination based on QIC

Supplementary file 7 – Collinearity analysis

Supplementary file 8 – Coefficients of novel PSMA-MRI combined nomogram and original MRI-based nomogram

|  | **MRI-only model** |
| --- | --- |
| **Intercept** | -3.065 |
| **PSA density** | 0.819 |
| **MRI**  No lesion  Organ-confined  EPE | 0.857  2.134 |
| **ISUP Grade**  Benign  1  2  3  4  5 | 0.393  1.109  1.494  1.790  2.295 |

|  | **PSMA-MRI model** |
| --- | --- |
| **Intercept** | -2.646 |
| **PSA density** | 0.430 |
| **MRI**  No lesion  Organ-confined  EPE | 0.267  1.413 |
| **ISUP Grade**  Benign/1  2  3  4  5 | 0.698  1.341  0.918  1.400 |
| **PSMA**  No lesion  Organ-confined  EPE | 0.512  0.951 |
| **PSMAtotal** | 0.543 |

Supplementary file 9 - Model performance on tracer level

68Ga-PSMA: AUC 0.78 (95% CI 0.73-0.83)
18F-PSMA: AUC 0.83 (95%CI 0.79-0.87)

Supplementary File 10. Diagnostic performance of the updated PSMA-MRI nomogram across predicted risk thresholds

**Development cohort**

801 lobes from 401 patients; 308 EPE-positive lobes (38%).

| **Threshold (%)** | **Sensitivity** | **Specificity** | **PPV** | **NPV** | **FN rate** | **Missed EPE (n/total EPE)** | **Below threshold, n (%)** | **EPE below threshold, n (%)** | **At/above threshold, n (%)** | **No EPE at/above threshold, n (%)** |
| --- | --- | --- | --- | --- | --- | --- | --- | --- | --- | --- |
| 10 | 0.97 (0.95–0.99) | 0.17 (0.14–0.20) | 0.42 (0.39–0.46) | 0.91 (0.83–0.95) | 0.03 | 8/308 | 90 (11%) | 8 (9%) | 711 (89%) | 411 (58%) |
| 15 | 0.93 (0.90–0.95) | 0.33 (0.29–0.37) | 0.46 (0.43–0.50) | 0.89 (0.83–0.92) | 0.07 | 21/308 | 183 (23%) | 21 (11%) | 618 (77%) | 331 (54%) |
| 20 | 0.90 (0.86–0.93) | 0.42 (0.38–0.47) | 0.49 (0.45–0.53) | 0.87 (0.83–0.91) | 0.10 | 30/308 | 238 (30%) | 30 (13%) | 563 (70%) | 285 (51%) |
| 30 | 0.81 (0.77–0.85) | 0.61 (0.57–0.66) | 0.57 (0.52–0.61) | 0.84 (0.80–0.88) | 0.19 | 57/308 | 360 (45%) | 57 (16%) | 441 (55%) | 190 (43%) |
| 40 | 0.71 (0.65–0.76) | 0.75 (0.71–0.78) | 0.64 (0.59–0.69) | 0.80 (0.77–0.84) | 0.29 | 90/308 | 459 (57%) | 90 (20%) | 342 (43%) | 124 (36%) |

**Validation cohort**

287 lobes from 144 patients; 69 EPE-positive lobes (24%).

| **Threshold (%)** | **Sensitivity** | **Specificity** | **PPV** | **NPV** | **FN rate** | **Missed EPE (n/total EPE)** | **Below threshold, n (%)** | **EPE below threshold, n (%)** | **At/above threshold, n (%)** | **No EPE at/above threshold, n (%)** |
| --- | --- | --- | --- | --- | --- | --- | --- | --- | --- | --- |
| 10 | 0.97 (0.90–0.99) | 0.17 (0.12–0.22) | 0.27 (0.22–0.33) | 0.95 (0.83–0.99) | 0.03 | 2/69 | 38 (13%) | 2 (5%) | 249 (87%) | 182 (73%) |
| 15 | 0.97 (0.90–0.99) | 0.37 (0.31–0.43) | 0.33 (0.27–0.39) | 0.98 (0.92–0.99) | 0.03 | 2/69 | 82 (29%) | 2 (2%) | 205 (71%) | 138 (67%) |
| 20 | 0.91 (0.82–0.96) | 0.47 (0.40–0.53) | 0.35 (0.29–0.42) | 0.94 (0.88–0.97) | 0.09 | 6/69 | 108 (38%) | 6 (6%) | 179 (62%) | 116 (65%) |
| 30 | 0.86 (0.75–0.92) | 0.67 (0.60–0.73) | 0.45 (0.37–0.54) | 0.94 (0.89–0.96) | 0.14 | 10/69 | 156 (54%) | 10 (6%) | 131 (46%) | 72 (55%) |
| 40 | 0.75 (0.64–0.84) | 0.80 (0.74–0.85) | 0.54 (0.44–0.64) | 0.91 (0.86–0.94) | 0.25 | 17/69 | 191 (67%) | 17 (9%) | 96 (33%) | 44 (46%) |

*A lobe is classified as at or above the threshold when the predicted probability of EPE equals or exceeds the stated value. Thresholds are presented for descriptive purposes, and no specific threshold is recommended. EPE = extraprostatic extension; PPV = positive predictive value; NPV = negative predictive value.*
